# Supplementary figures and images for: Proteomics reveals extensive phosphoregulation of outer kinetochore protein KNL1
Source: bioRxiv. 2026 Apr 3:2026.03.13.711714. Preprint. [Version 2] doi: 10.64898/2026.03.13.711714 (PMC13060184; doi:10.64898/2026.03.13.711714)

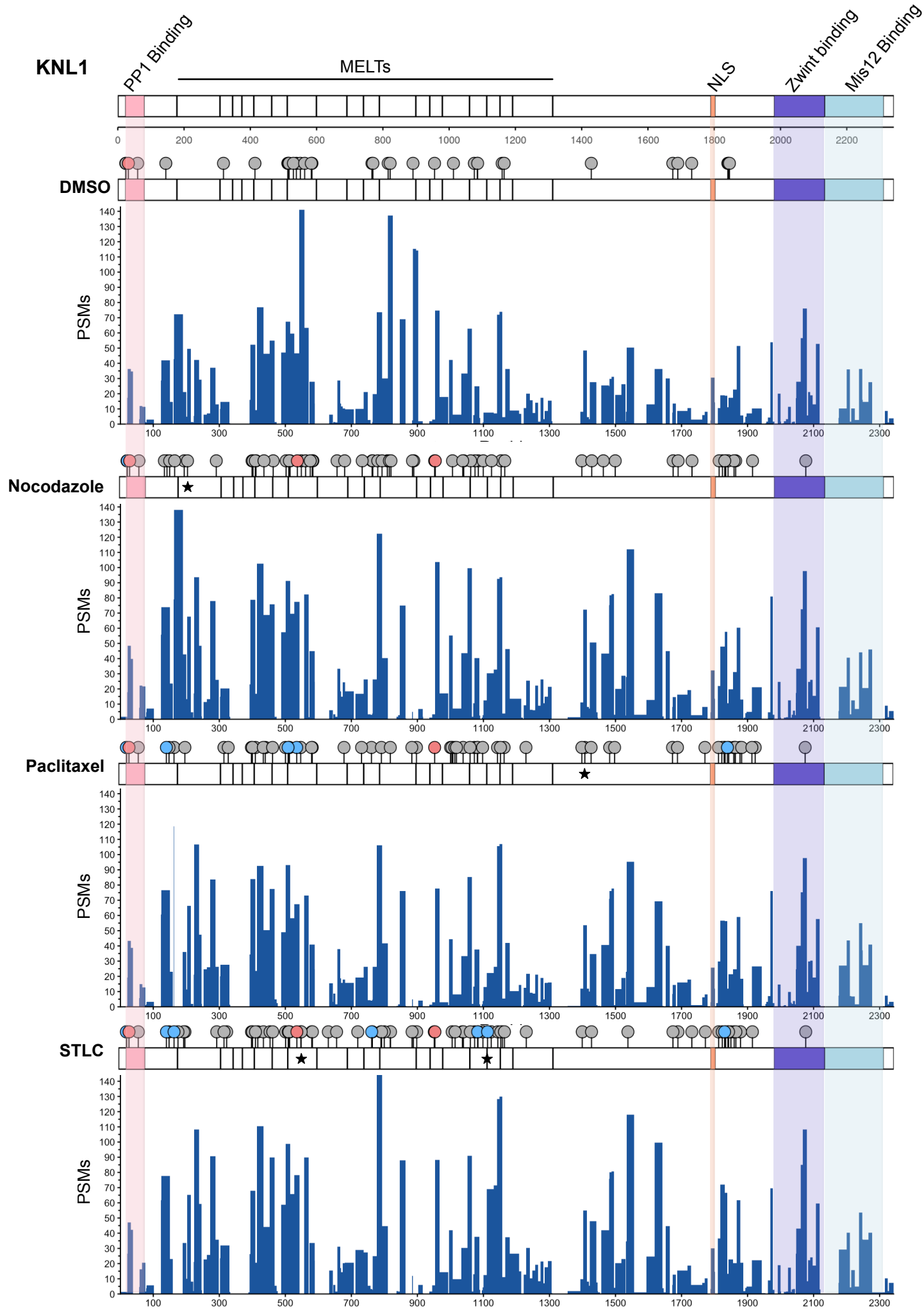

Supplement: Supplement 1 — Extended Data 1 Coverage maps of KNL1 illustrating average coverage for each condition across 3 mass-spectrometry experiments. Maps are aligned with corresponding phosphorylation schematics from Fig. 1D [file media-1.pdf]
